# Expanding Housing With Services in the United States: The Case of the Right Care, Right Place, Right Time Program

**DOI:** 10.1093/geroni/igaa057.1948

**Published:** 2020-12-16

**Authors:** Edward Miller, Pamela Nadash, Elizabeth Simpson, Marc Cohen

**Affiliations:** University of Massachusetts Boston, Boston, Massachusetts, United States

## Abstract

Older people living in congregate environments are obvious beneficiaries of supportive services. The potential for prevention is clear, particularly among low-income elders living in subsidized housing; it is this group that is at high risk for significant healthcare and other costs, and it is this group that suffers considerably from a fragmented healthcare system. The purpose of this presentation is to illustrate the potential of housing with services, drawing from evaluation of The Right Care, Right Place, Right Time (R3) initiative (R3) located in the Greater Boston area. The R3 program consists of two on-site wellness teams, including a wellness nurse and wellness coordinator. Each team is responsible for about 200 participants across two housing sites. Evaluation findings highlight the potential of housing with services for improving the health, quality of life, and access to health-related services and supports among seniors living independently in affordable housing, while reducing healthcare costs.

